# Cyanobacteria Under UV Radiation: General Insights into Stress Responses

**DOI:** 10.3390/ijms262210926

**Published:** 2025-11-11

**Authors:** Zofia Mazur, Ireneusz Ślesak

**Affiliations:** The Franciszek Górski Institute of Plant Physiology Polish Academy of Sciences, Niezapominajek 21, 30-239 Krakow, Poland

**Keywords:** cyanobacteria, ultraviolet radiation (UVR), reactive oxygen species (ROS), scytonemin (Scy), DNA repair, mycosporine-like amino acids (MAAs), space mission

## Abstract

One of the first organisms to appear on Earth was cyanobacteria, which carried out oxygenic photosynthesis. The oxygen they produced contributed to the ozone layer’s formation. However, before this happened, cyanobacteria had to cope with various forms of radiation, including ultraviolet radiation (UVR), that reached the surface of young Earth. Billions of years ago, before the Earth’s ozone layer formed, the planet was constantly exposed to intense UVR. This radiation, especially UVB and UVC, was strong enough to break down proteins and nucleic acids. Cyanobacteria have a variety of defence mechanisms that allow them to thrive under adverse conditions. These mechanisms include the avoidance of UVR through migration or mat formation, DNA repair, antioxidant enzyme activity, and biosynthesis of UVR-absorbing compounds. Although most of today’s dangerous UVR is absorbed by the ozone layer, future space exploration has led to a closer examination of the effects of UVR, especially UVC, on various organisms, including cyanobacteria. The flexibility of cyanobacteria to tolerate unfavourable conditions makes them potential candidates for future space exploration. This brief overview provides some information on the effects of UVR on cyanobacteria, the defence mechanisms of cyanobacteria against UVR, and the potential use of cyanobacteria in life-support systems in space missions.

## 1. Introduction

Today, the atmospheric ozone layer absorbs harmful ultraviolet radiation (UVR), preventing it from reaching the Earth’s surface and damaging living organisms by degrading proteins and DNA and forming reactive oxygen species (ROS). But this was not always the case. Billions of years ago, the atmosphere of the early Earth was very different and possibly similar to other planets that could be considered for human habitation, such as Mars. A key moment in the history of our planet was the Great Oxidation Event (GOE; ca. 2.4 to 2.1 billion years ago). The enormous increase in oxygen (O_2_) concentration during the GOE led to the formation of an oxygen-rich atmosphere in which life thrives today [1].

**Figure 1:**
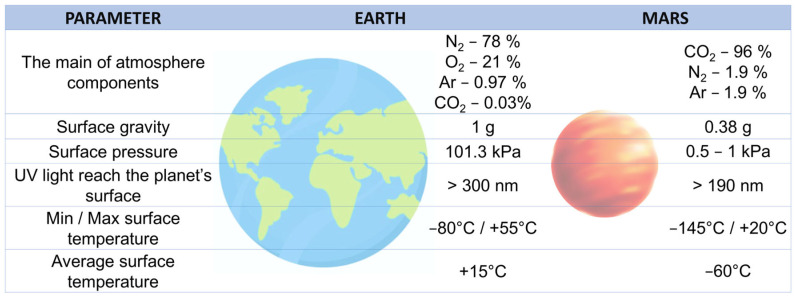
The comparison of Earth’s and Mars’s present-time environmental condition [10].

Cyanobacteria, which appeared on Earth ca. 3.5–2.8 billion years ago, were the first organisms to acquire the ability to perform oxygenic photosynthesis (OP), which enabled them to play a crucial role in this event [2,3,4]. Cyanobacteria also have a wide range of physiological properties and highly developed adaptive abilities. These characteristics enable them to survive in almost any condition, including hot springs, Arctic lakes, bare rocks, and even inside bare rocks [5,6]. Cyanobacteria have a remarkable tolerance to different types of radiation, for example, UVR and gamma radiation [7]. All of this makes them good candidates for terraforming—the process of altering the environment of a planet to make it more suitable for life. Scientists believed that by introducing such organisms to planets like Mars, which already has frozen water and carbon dioxide (CO_2_) on its surface, it might be possible to slowly enrich the environment with O_2_ and organic matter. Theoretically, this could prepare the ground for more advanced life forms or even make the planet habitable for humans in the distant future. However, this idea is still largely hypothetical (the environmental conditions on Mars differ from those on Earth—Figure 1) and research is focused on the use of these organisms in space mission support systems (e.g., the MELiSSA project, BOSS experiment, see below) [8,9]. Due to the great interest in this topic, this article presents the most important information about cyanobacteria, their defence mechanisms against UVR, and their potential use in future space missions.

## 2. UV Radiation and Its Influence on Cyanobacteria

UVR is divided into three categories: UVC (100–280 nm), UVB (280–315 nm), and UVA (315–400 nm). They differ in their biological activity and harmfulness—the shorter the wavelength, the greater the damage caused by UVR. Significantly, only a small amount of UVA (less than 7%) and UVB rays (<1%) reach the Earth’s surface. The rest, like UVC, is absorbed by the ozone layer [11]. In addition, artificially generated UVC is often used to disinfect water and other liquids, as well as various surfaces such as hospital rooms and shop shelves [12].

Exposure to UVR is toxic and can lead to the degradation or transformation of cellular components, ultimately resulting in the loss of their biological function. This process is referred to as a direct mechanism. On the other hand, indirect mechanisms take place when UVR is absorbed by extra- or intracellular compounds, which in turn promote the formation of ROS, such as hydrogen peroxide (H_2_O_2_), superoxide anion radicals (O₂·⁻), or hydroxyl radicals (HO·). Some of these ROS can diffuse and react with cellular components, causing damage beyond the site of their photoproduction [13,14,15,16].

***UVC*** is the most harmful form of UVR. In plants, it damages the photosynthetic electron transport chain in photosystem II (PSII) [17]. Recent studies have shown that UVC suppresses the expression of photosynthetic genes in cyanobacteria [18,19]. In addition, negative effects on the concentration of photosynthetic pigments (chlorophyll *a* (Chl *a*) and phycobiliproteins) were observed seven days after UVC irradiation [18]. UVC irradiation of *Chroococcidiopsis* sp. CCME 029 leads to cell lysis, the formation of cellular aggregates, genome damage, the accumulation of carotenoids, and a decrease in the maximum photochemical efficiency of PSII (Fv/Fm) [20]. Moreover, cyanobacteria exposed to UVC developed thick, protective envelopes that limited the penetration of UVC into the inner cell layers [20]. Interestingly, the average UVC doses required to inactivate 90% of *Synechocystis* sp. PCC 6803 and *Synechococcus* sp. PCC 7942 cells were 500 J m^–2^ and 400 J m^–2^, respectively [21,22]. Two of the nine *Nostoc* species, i.e., *N. sphaericum* ISB97 and *Nostoc* sp. ISB99, which inhabit areas with high levels of solar radiation showed 100% survival immediately after one hour of UVC irradiation (400 µW/cm^2^). UVC treatment reduced the content of photosynthetic pigments, while the activity of antioxidant enzymes was increased [23].

***UVB*** is the most dangerous UVR that reaches the Earth’s surface. It can cause direct damage to biological molecules, including the formation of thymine dimers and DNA strand breaks. UVB can also have an indirect effect on cells by generating ROS, e.g., by triggering lipid peroxidation [24]. Vass et al. estimated that ca. 75–80% of UVB-induced DNA damage consists of cyclobutane pyrimidine dimers (CPDs) [25]. The literature indicates that UVB negatively affects cyanobacteria in various ways, such as growth, photosynthetic activity, nitrogen metabolism, the disruption of cell motility, and morphological changes in filamentous cyanobacteria (reduced filament length) [24]. In addition, UVB exposure leads to a reduction in the content of photosynthetic pigments and to changes in the composition of phycobiliproteins [24,26]. Exposure to UVB results in a decline in the levels of saturated, monounsaturated, and polyunsaturated fatty acids, as well as proteins and certain amino acids, in *Synechococcus leopoliensis* [27].

***UVA*** is the main UVR of the sunlight that reaches the surface of our planet and affects cells through intracellular ROS formation [24,28]. The overproduction of ROS leads to single- or double-strand breaks in DNA [29]. UVA also induces changes in nitrogen metabolism, photosynthetic pigment content, and phytoplankton primary production [30]. There are also some negative effects on photosynthesis, e.g., the inhibition of the photosynthetic electron transport chain, modulation of quinone B (Q_B_)-binding affinity and degradation of the D1 and D2 proteins of PSII [31]. In the case of *Anabaena siamensis* TISTR-8012, exposure to photosynthetically active radiation (PAR) in conjunction with UVA and UVB radiation (PAB) induced the degradation of cyanobacterial filaments and diminished cell differentiation, resulting in a decline in the number of heterocysts and akinetes [32].

The studies on cyanobacteria species with and without heterocysts showed a decrease in Chl *a* and an increase in carotenoid (Car) content after exposure to PAB, regardless of whether the strain had heterocysts or not. A decrease in phycocyanin (PC) content was observed in cyanobacteria without heterocysts, while PC was more stable in species with heterocysts. This indicates a better adaptation of heterocystic cyanobacteria to PAB irradiation [33]. A metabolomic study of cyanobacteria irradiated with UVA and UVB rays revealed that approximately 7% of features were exceptional for the metabolomic profiles of cyanobacteria before UV irradiation. After UVR irradiation, 9% of the profiles were unique. Several groups of compounds were identified, e.g., mycosporine-like amino acids (MAAs), microcystins (MCs), and microginins. The levels of most metabolites belonging to, for example, pseudospumigins and microginins, decreased after exposure to UVR. Conversely, MAAs, MC, and cyanopeptolins levels increased [34].

Zhu et al. [17] studied the effect of UVA and UVC synergies in controlling harmful cyanobacterial blooms and indicated that UVA irradiation (0.085 J cm^−2^) before UVC irradiation (0.085 J cm^−2^) inhibits the growth of *Microcystis aeruginosa* and initiates a regulated cell death (RCD), which then leads to cell lysis. They also point to the synergistic inhibition of photosynthesis by UVA and UVC irradiation: UVA irradiation damages photosystem I (PSI), while the combination with UVC also causes the destruction of PSII [17].

## 3. Cyanobacterial Adaptation Mechanisms Against UV Irradiation

**Figure 2:**
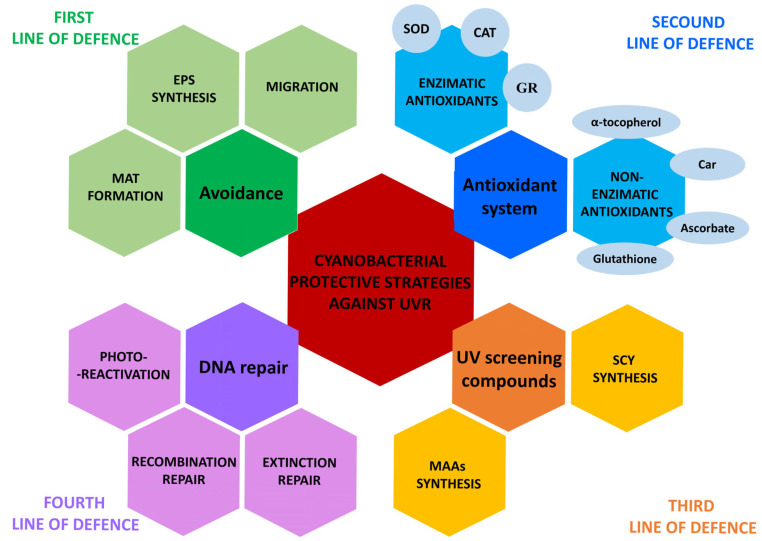
The lines of defence that cyanobacteria use against UVR. EPS—exopolysaccharides, CAT—catalase, SOD—superoxide dismutase, GR—glutathione reductase, Car—carotenoids, MAAs—mycosporine-like amino acids, and Scy—scytonemin.

During the Archean eon (ca. 3.5–2.8 billion years ago), cyanobacteria thrived under conditions of intense UVR. Without a protective ozone layer, they absorbed much higher doses of UVR, including UVC, than organisms are exposed to today [35,36]. Therefore, cyanobacteria had to have adaptive mechanisms to withstand these stressful conditions. Four defence mechanisms (Figure 2) can be distinguished: (1) avoidance (e.g., migration and mat formation), (2) antioxidant systems, (3) UV-screening compounds, and (4) DNA repair [6,16].

### 3.1. Avoidance

The first line of defence is to avoid areas with excessive UVR and PAR exposure (Figure 2). This is usually achieved by the formation of mats, the migration to lower-light environments, or the production of extracellular polysaccharides [37]. The mats can contain many different species of cyanobacteria (both filamentous and unicellular forms) as well as other microorganisms that often live in symbiosis. Their synchronised movement within the mat provides the resources necessary for survival [38,39]. Some organisms have been found in the deeper layers of the mats, which provide protection from UVR and short-wave light in the PAR range [40,41]. An example of this is a mat dominated by the filamentous cyanobacteria *Lyngbya* cf. *aestuarii* and *Microcoleus chthonoplastes*, which were observed near a subtropical mangrove system. The upper layer contained *L. aestuarii*, which produces scytonemin (Scy). *M. chthonoplastes* lacks this photoprotective compound and prefers the deeper-mat area to avoid high light intensities. It has been suggested that the Scy of *Lyngbya* represents a photoprotective mechanism for *M. chthonoplastes* [42].

Organisms that form mats in areas with variable light intensity often have the ability to migrate. In response to environmental conditions, they move towards (positive phototaxis) or away from (negative phototaxis) light sources [41,43]. Intense sunlight, for example, caused *Oscillatoria* sp. and *Spirulina* cf. *subsalsa* to migrate downwards. This migration did not take place in the dark [44]. Moreover *Synechocystis* sp. PCC 6803 exposed to UVA shows negative phototaxis [45]. It is reported that cyanobacteria exposed to high intensities of PAR and UVR moved as far away from the light source as possible, indicating a defence mechanism against harmful radiation [46]. There is also some information on the potential receptors involved in photoprotection against UVR. Moon et al. [47] demonstrated that the *cph2* gene, which encodes the cyanobacterial phytochrome, is involved in inhibiting migration towards the UVA radiation source (positive phototaxis). Consequently, *Synechocystis* sp. PCC 6803, which lacks the Cph2 protein due to the inactivation of the gene (*cph2*), showed positive phototaxis [47]. A signal transduction system that is activated by UVA radiation and is responsible for negative phototaxis, has also been proposed. This system consists of the UirR/UirS proteins together with the LsiR protein [48]. One possible mechanism is that the UirR/UirS protein complex, which is attached to the plasma membrane, responds to UVA radiation. UVA causes the UirR protein to detach from UirS. The released UirR then binds to the promoter of the *lsiR* gene (slr1214). This process initiates the expression of the signal transduction regulator LsiR, which leads to negative phototaxis [48]. The table below summarises key species, the light bands (PAR and/or UV) that trigger phototactic responses, photoreceptors, and ecological contexts (Table 1).

### 3.2. Antioxidant System

The antioxidant system is the second line of defence against UVR, which includes non-enzymatic (e.g., carotenoids, ascorbate (vitamin C), α-tocopherol (vitamin E), and reduced glutathione) and enzymatic antioxidants, e.g., superoxide dismutase (SOD), catalase (CAT), various peroxidases, and glutathione reductase (GR) [16] (Figure 2).

Carotenoids (Car) are antioxidants that protect cells from ROS and dissipate excess light energy as heat. They also exert a photoprotective function against UVR. Exposure to UVR increases the content of certain Car in studied cyanobacterial species [58,59,60,61]. Rastogi et al. (2015) analysed the literature and demonstrated the role of Car (canthaxanthin, echinenone, myxoxantophyll, and zeaxanthin) in protecting against damage caused by oxidative factors, such as intense PAR or UVR [37].

Superoxide dismutases (SODs) belong to the metalloproteins and carry out the reaction of dismutation of superoxide anion radicals (one of the ROS) leading to the formation of less-reactive compounds, i.e., O_2_ and H_2_O_2_. Cyanobacteria belonging to the *Nostoc* group, exposed to UVA and UVB rays and dehydration, showed increased expression of the *wspA* and *sodF* genes, which encode water-stress proteins (WspA) and Fe-SOD (SodF), respectively. This suggests that these proteins are involved in protecting the cells of *Nostoc commune* DRH1 from UVR and dehydration [62]. In addition, an increase in SOD activity was observed in *Plectonema boryanum* under UVB irradiation [63] as well as in *Fischerella* sp. strain HKAR-14 [64] and *Spirulina subsalsa* HKAR-19 [65] under PAR in conjunction with UVA (PA) or PAB.

Catalases (CATs) are another group of enzymes that indirectly protect cells from UVR by converting H_2_O_2_ to H_2_O and O_2_ [66,67]. The increase in CAT activity in the cyanobacteria *Microcoleus vaginatus* under UVB irradiation, *Nostoc* sp. HKAR-2 exposed to UVB or PAR in conjunction with UVB (PB) irradiation, and *Fischerella* sp. strain HKAR-14 under PA or PAB irradiation has been reported in the literature [64,68,69]. Yan et al. (2024) observed that high temperatures together with UVR irradiation induced higher SOD and CAT activity [70].

Ascorbate peroxidases (APXs) catalyse the reduction of H_2_O_2_ to water using ascorbate. APX activity was found to be positively correlated with exposure time in *Spirulina subsalsa* HKAR-19 exposed to UVR [65]. In addition, SOD and APX activity increased in *Nostoc spongiaeforme* and *Phormidium corium* in response to UVR [71]. Studies of the effect of UVB on the activity of CAT, SOD, and APX in two *Nostoc* species showed a significant increase in the activity of these enzymes [72]. After exposure to UVR, different species of cyanobacteria exhibited varying degrees of induction of antioxidant enzyme activity. This activity could regulate the survival rate of cyanobacteria under unfavourable environmental conditions [72]. However, the results concerning APX activity in cyanobacteria should be treated with caution, as cyanobacteria generally lack APX genes in their genomes and, consequently, APX proteins, although APX-like activity has been identified in some species [73,74].

### 3.3. UV-Absorbing Compounds

Cyanobacteria have also developed effective protective strategies based on the synthesis of UV-absorbing compounds. The most important of these are mycosporine-like amino acids (MAAs) and scytonemin (Scy). MAAs are a group of water-soluble compounds localised in an exopolysaccharide (EPS) matrix that surround the cells of many cyanobacteria [75,76]. MAAs exhibit photostability in both fresh and salt water, and their UV absorption maxima are between 310 and 362 nm, depending on the structure of the compound. They are also capable of realising the absorbed UVR in the form of heat without generating ROS, which can be toxic to cells [61,77]. The amount of MAAs in cyanobacteria increases when they are exposed to excess light, UVR, heat stress, or increased salinity [78,79,80]. The biosynthesis of MAAs is strongly influenced by environmental factors, especially UVR. The induction of their synthesis under UV stress suggests their possible role in photoprotection. Their high concentration can make the cell up to 25% more resistant to UVR with a wavelength of 320 nm [81,82]. Interestingly, the UVB photoreceptor that induces the synthesis of MAAs was found in *Chlorogloeopsis* PCC 6912 [83].

Another compound mentioned above that can absorb UVR and dissipate it as heat is Scy [76]. However, it mainly absorbs UVA and reduces its radiation at the cell surface by 90% [84]. Scy is a yellow-brown, lipid-soluble pigment localised in the EPS matrix. Scy occurs in two forms: oxidised (yellow fuscochlorin) and reduced (red fuscorhodin). It demonstrates high stability under various conditions, such as one-hour exposure to UVB radiation, high temperatures (60 °C), or strong oxidising agents (0.25% H_2_O_2_) [85]. Scy shows a radical-scavenging effect, such as 2,2-diphenyl-1-picrylhydrazyl (DPPH) or 2,2′-azinobis(3-ethylbenzothiazoline-6-sulfonic acid) (ABTS) [85]. UVA and other stress factors, such as salinity and desiccation, stimulate Scy biosynthesis [86]. Dillon et al. (2002) reported an increase in Scy content in response to oxidative and temperature stresses in the presence of UVA in *Chroococcidiopsis*, while biosynthesis was inhibited after exposure to salt [87]. In contrast to *Chroococcidiopsis*, salt induced the synthesis of Scy in *Lyngbya aestuarii* [88]. Madrahi and Naeimpoor have compiled studies indicating an increase in Scy in various species of cyanobacteria, such as *Anabaena* sp., *Aphanocapsa* sp., *Chlorogloeopsis* sp., *Chroococcus* sp., *Calothrix* sp., *Diplocolon* sp., *Scytonema* sp., and *Tolypothrix* sp. after exposure to UVR and other abiotic stresses [89].

The role of temperature in Scy biosynthesis is significant, especially when cells are exposed to elevated temperatures in addition to UVA. An increase in temperature led to higher Scy content in *Chroococcidiopsis*, while UVA irradiation further and markedly increased Scy levels. Thus, there was a synergistic interaction between elevated temperature and UVA irradiance on Scy biosynthesis [87]. It was also found that increased temperature enhanced *M. aeruginosa* sensitivity to UVR by reducing PSII maximum photochemical efficiency (Fv/Fm). *M. aeruginosa* grown at high temperatures showed a lower PSII repair rate, sustained induction of non-photochemical quenching (NPQ), and increased MCs content during UVR exposure [70].

### 3.4. DNA Repair

The next line of defence is activated when other protective mechanisms fail and UVR penetrates the cells. Organisms become more resistant to harmful conditions such as UVR if they have DNA repair mechanisms. The most important of these include photoreactivation, extinction repair, and recombination repair. Photoreactivation is a light-dependent process carried out by photolyase and is the most abundant repair mechanism in cyanobacteria [16]. There are two types of photolyases that repair different substrates: CPD photolyases (which repair cis-syn-cyclobutane pyrimidine dimers; CPDs) and (6-4)-photolyases (which repair pyrimidine-pyrimidone (6-4) photoproducts; (6-4)-PPs). It was shown that the PhrA protein from *Synechocystis* sp. PCC 6803, which is encoded by the *phrA* gene, belongs to the family of CPD photolyases. Mutations in this gene have been linked with the increased sensitivity of cells to UVB and UVC [90]. The *Synechocystis* sp. PCC 6803 mutants with photolyase deficiencies are unable to repair UVB-induced DNA damage, which has been associated with reduced PSII activity and decreased levels of D1 protein [25].

An example of DNA extinction repair is nucleotide excision repair (NER), which is involved in the repair of various types of DNA damage, e.g., CPDs, (6-4)-PPs, and DNA intrastrand crosslinks [91,92]. The genes of the *uvrABCD* repair system, which are responsible for the NER mechanism, have been shown to be present in the genomes of cyanobacteria. However, *uvrA* and *uvrB* are not part of an operon, as is the case in *Escherichia coli*. It has also been observed that some DNA repair genes (*phr* and *recN*) are linked to *uvrA*, *uvrB*, *uvrC*, or *uvrD* genes [93]. Interestingly, some cyanobacteria have two copies of the *uvrC* and *uvrD* genes. It is hypothesised that the second copy of *uvrC* may be a gene encoding a Cho protein that is the homologue of UvrC, which was first described in *E. coli* [94]. Furthermore, the overexpression of, *uvrB*, *uvrC*, and *uvrD* genes was observed in *Arthrospira* PCC 8005 exposed to gamma radiation [95].

## 4. The Ecological Context of UV Treatment on Cyanobacteria

UV irradiation, particularly UVB and UVC, damages cyanobacterial cells, leading to various ecological side effects, such as the release of intracellular toxins like microcystins (MCs) into the surrounding water. In addition, other secondary metabolites, such as cyanopeptolins, may increase under UV stress, potentially contributing to the overall toxicity and ecological impact of treated waters [96,97,98,99]. UV acts as an environmental stressor, modulating the expression of genes involved in MC biosynthesis (e.g., *mcyA*, *mcyD*, and *mcyE*). Under UVB, evidence shows the upregulation of MC synthesis genes during stress periods, suggesting a stress-induced signalling mechanism [99]. However, prolonged or intense UV exposure can also reduce the abundance of toxin-producing strains and decrease total toxin content due to cell death [96]. UVC can also degrade MCs, but the efficiency and toxicity of the by-products are not fully characterised, and there is a risk of secondary pollution if degradation is incomplete [98,100].

Metabolomic analyses indicate that MC production is linked to stress adaptation. Therefore, increased toxin biosynthesis under UV is likely an adaptive response to oxidative and light stress, rather than simply a by-product of cell damage [97,99]. It has also been suggested that MC plays a role in stabilising proteins (e.g., RubisCO) by binding to their cysteine residues under high-light conditions [101]. For this reason, MC might be involved in the acclimation of cyanobacteria to excess light stress [102].

UV-induced cell lysis also increases the release of organic matter, including both dissolved and particulate fractions. This organic matter is more labile and bioavailable, potentially fuelling heterotrophic bacterial growth. Enhanced organic matter release can increase the pool of substrates for microbial respiration, raising oxygen demand and potentially contributing to hypoxic conditions in water reservoirs [100,103].

In summary, the upregulation of toxin biosynthesis under UV is primarily a stress adaptation, but the net ecological impact depends on the treatment intensity, environmental context, and the fates of released toxins and organic matter.

## 5. The Potential Use of Cyanobacteria in Biological Life-Support Systems (BLSSs) and as Extraterrestial Planet Terraforming Agents

Cyanobacteria are proposed as potential candidates for use in biological life-support systems (BLSSs). Compared to other organisms, cyanobacteria have simpler nutrient requirements and are highly tolerant to extreme conditions, including UVR, desiccation or high CO_2,_ making them more resilient for extraterrestrial or harsh environments. Moreover, the prokaryotic nature of cyanobacteria means that genetic manipulations are easier in them than in many microalgae. There are well-developed genetic tools for model cyanobacterial strains [10,104,105]. In addition, cyanobacteria were the first species capable of oxygenic photosynthesis, which may have contributed to the emergence of the GOE. It is hypothesised that cyanobacteria could be crucial for initiating a similar oxygenation process on Mars or other extraterrestrial planets during terraforming. They could also be used to generate O_2_ and remove CO_2_ in life-support systems during space missions.

It is known that cyanobacteria can develop a local ecosystem and support heterotrophic communities through rock colonisation, nitrogen (N_2_) fixation, and the production of organic compounds. For this reason, cyanobacteria can be used as a source of organic compounds to cultivate other microorganisms, such as heterotrophic bacteria, which could potentially be used for the production of pharmaceuticals, food, various industrially useful chemicals or biomaterials, as well as for the leaching of metals that may be valuable for supporting a future Mars mission [105]. Of particular interest in this context are diazotrophic cyanobacteria, that are able to convert atmospheric nitrogen into ammonia (NH_4_^+^), which can then be absorbed by plants and serve as a natural fertiliser. In plant cultures, however, cyanobacteria are not used as the sole source of nutrients, but to balance mineral nutrition or promote plant growth [106]. Interestingly, Arai et al. [107] has observed that it is possible to culture *Nostoc* sp. HK-01 on a Martian regolith simulant for ca. 140 days. Apart from atmospheric gases, no other nutrient was supplied [107]. It is also reported that the Brown–Stanford iGEM team conducted an experiment with the nitrogen-fixing engineered cyanobacterium *Anabaena* sp. PCC 7120, producing sucrose, which was then used as an energy (carbon) source to cultivate *Bacillus subtilis* (https://2013.igem.org/Team:Stanford-Brown/Projects/EuCROPIS (accessed on 1 October 2025).

In summary, cyanobacteria strains with exceptional resilience to Martian conditions (Figure 1), such as desiccation, radiation (including UVR), and perchlorate toxicity, have recently been described [10,105]. Their ability to utilise in situ resources, such as regolith and atmospheric Martian gases, has also been examined. In particular, cyanobacteria from the genera *Chroococcidiopsis*, *Anabaena*, *Nostoc*, and *Arthrospira* have emerged as leading candidates, each offering unique advantages for space-based biotechnologies. The most promising cyanobacteria species for terraforming the surface of Mars and for use in BLSSs are listed in Table 2.

## 6. Cyanobacteria in Space Mission Experiments

In recent years, space agencies such as National Aeronautics and Space Administration (NASA) and the European Space Agency (ESA) have become increasingly interested in the use of microorganisms such as bacteria, cyanobacteria, and microalgae as part of their research into sustainable life-support systems. One reason for the development of BLSSs is the high cost of delivering resources, such as water and oxygen, into space. The longer a human space mission lasts, the more expensive it becomes [121,122].

Experiments are already being carried out on the International Space Station (ISS) and in Mars-like environments on Earth. Scientists are studying how effectively these microorganisms can grow and function under space or simulated Martian conditions, which are characterised by intense radiation, high vacuum, microgravity, or extreme temperatures [123] (Figure 1). One example of such experiments is the Micro-Ecological Life-Support System Alternative (MELiSSA) project.

**Figure 3:**
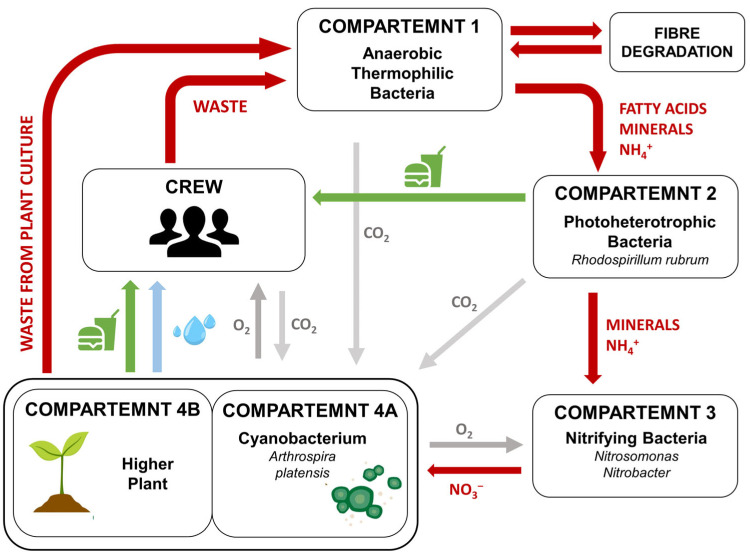
The scheme of the MELiSSA loop concept according to Lasseur et al. (2010) [9]. Five compartments can be distinguished to perform various functions: 1 and 2—degradation of organic waste by microorganisms; 3—nitrification process by nitrifying bacteria; 4A and 4B—edible materials, O_2_ production, and air revitalisation by the cyanobacteria and plants; and the crew compartment. The meaning of the different colours of arrows is as follows: red–waste degradation, green and blue–food and water production, respectively; grey–gas exchange in the MELiSSA loop.

The idea of the MELiSSA project was to create a regenerative, natural ecosystem-orientated support system for future long-term human space missions. The project consists of five compartments that fulfil different functions (Figure 3) [9]. The cyanobacterial and plant compartment is an important part of the MELiSSA loop because it provides the elements necessary for life, including CO_2_ fixation, O_2_ and drinking water production, and, most importantly, food production [9]. *Arthrospira* sp., an edible cyanobacterium, was used in the MELiSSA circuit due to its exceptional nutritional value. It is also very easy to cultivate and can absorb CO_2_ more efficiently compared to certain plants [124].

On the other hand, a valuable experiment, although not focused on space mission support systems, was the Biofilm Organisms Surfing Space (BOSS) experiment conducted on the ISS during the EXPOSE-R2 mission from 2014 to 2016. The aim of BOSS was to test whether microorganisms in the form of biofilms are better suited to long-term survival in space and under simulated Martian conditions than planktonic microorganisms. The space experiment was carried out with various microorganisms, e.g., (1) *Deinococcus geothermalis*—a polyextremophilic bacterium that is highly resistant to desiccation and radiation, (2) *Gloeocapsa* sp.—cyanobacteria from a previous experiment of the EXPOSE-E space mission, and (3) *Chroococcidiopsis* spp.—desert cyanobacteria [8]. Three different strains of *Chroococcidiopsis* spp. showed less DNA damage and a higher survival rate in biofilm than in the form of planktonic cells after exposure to Martian and space-like conditions. In addition, the photosynthetic pigments were better preserved in the lower layer than in the upper layer of the cells in the biofilm [125]. Wadsworth et al. (2019) reported that cell aggregates of *Gloeocapsa* sp., which do not occur in the form of structured biofilms, provide better protection against the stressful conditions in space compared to planktonic cells [126]. All these data support the hypothesis that bacteria, including cyanobacteria, in the form of biofilms have better chances of survival under the harsh environmental conditions in space and on Mars compared to planktonic cells.

## 7. Conclusions

Due to their diverse adaptation mechanisms and adaptability to various stress conditions, cyanobacteria can colonise a range of aquatic and terrestrial habitats, which makes them an interesting topic for future research. In addition, some cyanobacteria are resistant to various radiations (e.g., UV and gamma radiation) and have adaptive mechanisms that protect them from excessive UV radiation, e.g., avoidance (migration and mat formation), DNA repair, the presence of photoprotective compounds (Car, Scy, MCs, and MAAs), and antioxidant enzyme activity (SOD and CAT).

Most studies focus on UVA and UVB rays, which reach the Earth’s surface in small quantities. However, the possibility of exploring space and terraforming Earth-like planets in the future requires a change in direction in research, focusing instead on the effects of cosmic radiation and UVC-enriched conditions on living organisms on Mars and in space. This is already happening, for example, in the MELiSSA and BOSS experiments. However, in order to best prepare humans for the exploration of extraterrestrial planets such as Mars, future BLSS programmes must be further developed.

## Figures and Tables

**Table 1:** Selected cyanobacteria and their phototactic responses.

Species/Strain	Light	Phototactic Direction	Photoreceptors	Ecological Context	References
*Synechocystis* sp. PCC 6803	red, green	positive	TaxD1 (PixJ1), PixD	freshwater, biofilms, fluctuating light	[45,48,49,50,51,52]
	blue, UV	negative or inhibited	UirS, UirR (cyanobacteriochromes, CBCR), Cph2 (cyanobacterial phytochrome)		
*Synechococcus* OS-B′	UVA, blue, red, green	positive	PixJ, UirS	hot springs mats (50–55 °C)	[53]
*Synechococcus elongatus* UTEX 3055	blue, green	both (bidirectional)	PixJSe (CBCR)	soil, biofilms	[54,55]
*Phormidium lacuna*	broad spectrum (PAR)	positive (weak light), negative (strong light)	CphA (cyanobacterial phytochrome), PixJ (CBCR), PSII	filamentous, biofilms	[56,57]

**Table 2:** Cyanobacteria suitable for BLLSs and terraforming Mars and Mars-like environments.

Species/Strains	Key Features	References
*Chroococcidiopsis* sp. CCMEE 029 *Chroococcidiopsis* sp. CCMEE 029 P-MRS	resistance to desiccation, temperature fluctuations, UVR, and perchlorate, nutritional value for heterotrophic bacteria	[108,109,110,111,112]
*Anabaena* sp. PCC 7938	diazotrophy and rock-leaching abilities, as well as tolerance to perchlorates, feedstock for other organisms	[113,114,115,116]
*Desmonostoc muscorum* (*Nostoc muscorum*) UTAD N213 *Desmonostoc* sp. *Nostoc* sp. FACHB 892	resistance to desiccation and Mars-like conditions, food source	[117,118,119]
*Arthrospira* sp. PCC 8005 *Arthrospira platensis*	resistance to ionising radiation, food source	[117,120]

## Data Availability

No new data were created or analysed in this study.

## Abbreviations

The following abbreviations are used in this manuscript:

GOE	Great Oxidation Event
UVR	ultraviolet radiation
ROS	reactive oxygen species
MAAs	mycosporine-like amino acids
Scy	scytonemin
OP	oxygenic photosynthesis
MELiSSA project	Micro-Ecological Life-Support System Alternative project
BOSS experiment	Biofilm Organisms Surfing Space experiment
PQ	plastoquinone
Chl *a*	chlorophyll *a*
PSII	photosystem II
Fv/Fm	maximum photochemical efficiency of PSII
CPDs	cis-syn-cyclobutane pyrimidine dimers
(6-4)-PPs	pyrimidine-pyrimidone (6-4) photoproducts
NER	nucleotide excision repair
EPS	exopolysaccharides
PAR	photosynthetically active radiation (PAR)
PB	photosynthetically active radiation (PAR) in conjunction with UVB (PAR+UVB)
PA	photosynthetically active radiation (PAR) in conjunction with UVA (PAR+UVA)
PAB	photosynthetically active radiation (PAR) in conjunction with UVA and UVB (PAR+UVA+UVB)
Car	carotenoids
SOD	superoxide dismutase
CAT	catalase
GR	glutathione reductase
WspA	water-stress proteins
MC	microcystin
BLSSs	biological life-supported systems
ESA	European Space Agency
NASA	National Aeronautics and Space Administration
ISS	International Space Station
CBCR	cyanobacteriochrome
RubisCO	ribulose-1,5-bisphosphate carboxylase/oxygenase
